# Bilateral Adrenal Calcifications as an Imaging Clue to Wolman Disease in Early Infancy: A Case Report

**DOI:** 10.7759/cureus.103790

**Published:** 2026-02-17

**Authors:** Juan C Niño, Camilo A Caicedo, Jaime Cárdenas

**Affiliations:** 1 Radiology, Pontifical Javierian University, Bogota, COL; 2 Radiology, Hospital Universitario San Ignacio, Bogota, COL; 3 Radiology, Los Cobos Medical Center, Bogota, COL

**Keywords:** adrenal calcifications, cholesteryl ester storage disease, imaging findings, pediatric genetics, wolman disease

## Abstract

Wolman disease is a rare autosomal recessive lysosomal storage disorder caused by mutations in the LIPA gene, resulting in lysosomal acid lipase (LAL) deficiency and subsequent accumulation of triglycerides and cholesterol esters in multiple organs. We report the case of a two-month-old female infant with an insidious clinical course characterized by vomiting, postprandial abdominal distension, diarrhea, and failure to thrive, associated with hepatomegaly. Laboratory evaluation revealed markedly reduced total cholesterol, low-density lipoprotein, and high-density lipoprotein levels with elevated triglycerides.

Abdominal ultrasound demonstrated hepatosplenomegaly with diffuse increased hepatic echogenicity consistent with steatosis and bilateral adrenal enlargement with coarse echogenic foci producing posterior acoustic shadowing, suggestive of adrenal calcifications. These findings were confirmed on contrast-enhanced abdominal computed tomography, which showed the characteristic adreniform preservation of this disease. Given the suspicion of a lysosomal storage disorder, genetic testing identified a homozygous nonsense mutation in LIPA, and enzymatic analysis confirmed markedly reduced LAL activity, establishing the diagnosis of Wolman disease. Enzyme replacement therapy was initiated, with a favorable clinical response. This case highlights the critical role of imaging findings, particularly bilateral adrenal calcifications with preserved morphology, in raising early suspicion of Wolman disease and facilitating timely diagnosis and treatment.

## Introduction

Wolman disease is a rare autosomal recessive lysosomal storage disorder caused by pathogenic mutations in the LIPA gene, which encodes lysosomal acid lipase (LAL), an essential enzyme for the intracellular hydrolysis of triglycerides and cholesteryl esters. The LIPA gene is located on chromosome 10q23.2-q23.3 and consists of 10 exons [1,2]. Mutations resulting in markedly reduced or absent enzymatic activity are specifically associated with Wolman disease. Severe LAL deficiency leads to progressive accumulation of neutral lipids within lysosomes, predominantly affecting the liver, spleen, small intestine, and adrenal glands [3].

Clinically, Wolman disease typically presents during early infancy with nonspecific manifestations, including failure to thrive, vomiting, chronic diarrhea or steatorrhea, and progressive hepatosplenomegaly, often evolving toward liver failure [4-6]. Laboratory findings commonly reveal a characteristic lipid profile with hypertriglyceridemia, elevated low-density lipoprotein (LDL) cholesterol, and reduced high-density lipoprotein (HDL) cholesterol, accompanied by elevated liver transaminases [4]. Due to its nonspecific clinical presentation, the diagnosis may be delayed or initially misattributed to more common gastrointestinal or metabolic disorders.

Imaging plays a crucial role in raising early diagnostic suspicion. A hallmark radiologic feature of Wolman disease is bilateral adrenal enlargement with parenchymal calcifications while preserving the adreniform morphology, a finding present in approximately half of reported cases. Furthermore, the preservation of the adreniform morphology allows differentiation from other more common adreniform calcifications, such as adrenal hemorrhage or infection. Additional imaging findings of this pathology are hepatomegaly, hepatic steatosis, ascites, and intestinal wall thickening [5,7,8]. Recognition of these characteristic imaging patterns can prompt targeted biochemical and genetic testing, enabling earlier diagnosis and initiation of disease-modifying therapies. This report describes a case of Wolman disease in a young infant, emphasizing the diagnostic value of imaging findings in the early identification of this rare but life-threatening condition.

## Case presentation

A two-month-old female infant presented with an insidious clinical course characterized by vomiting, postprandial abdominal distension, diarrhea, and failure to thrive. Physical examination revealed erythematous plaques in skin folds and hepatomegaly. The patient was initially managed for suspected cow’s milk protein allergy, without clinical improvement. Laboratory investigations demonstrated markedly low total cholesterol, LDL, and HDL levels, with elevated triglycerides (Table 1).

**Table 1:** Lipid profile LDL: low-density lipoprotein; HDL: low-density lipoprotein

Total cholesterol (mg/dL)	LDL (mg/dL)	HDL (mg/dL)	Triglycerides (mg/dL)
297.4; reference range: 140-200 mg/dL	239; reference range: 0-150 mg/dL	8; reference range: <60 mg/dL	251; reference range: 35-150 mg/dL

**Figure 1:**
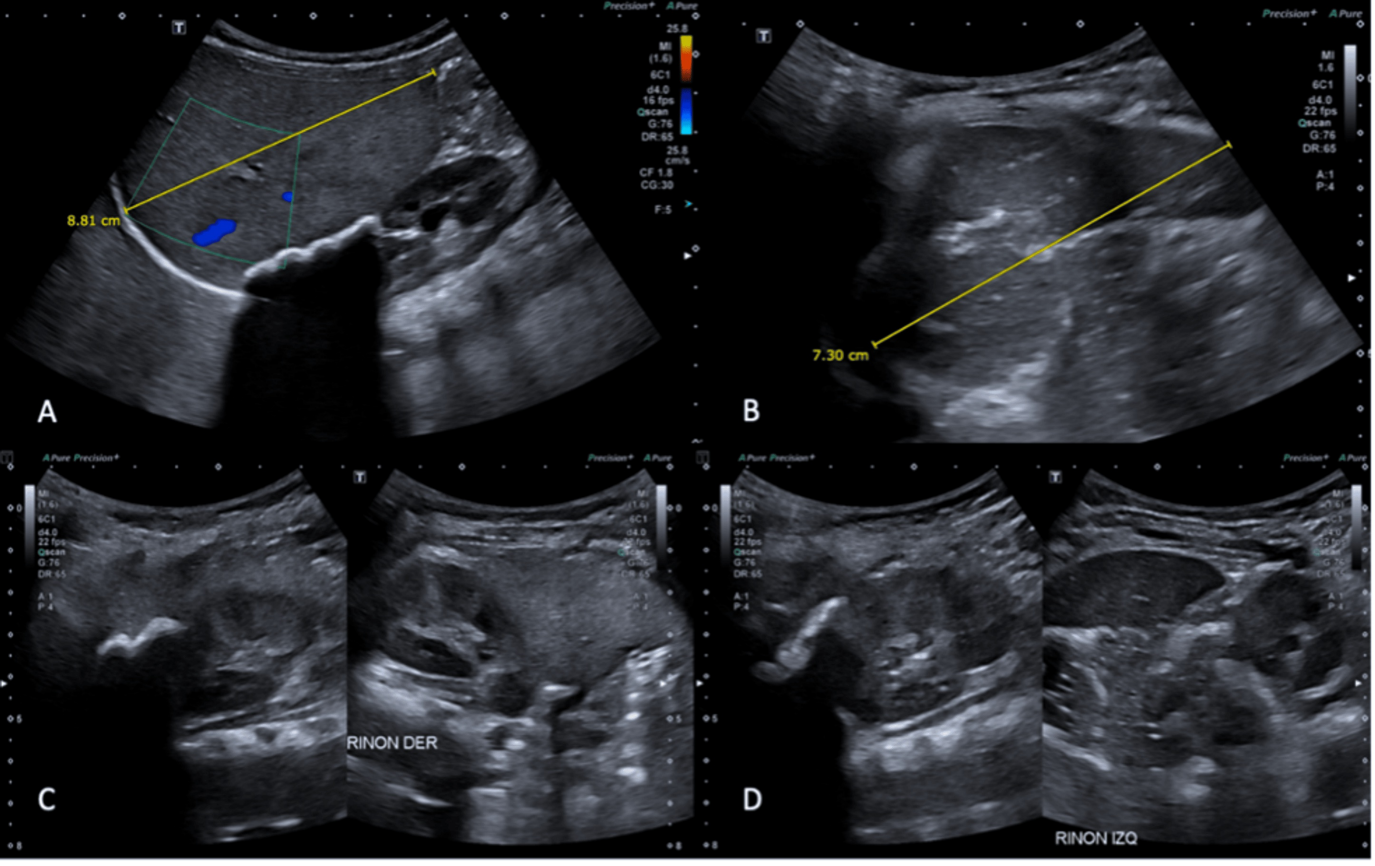
Selected images from a complete abdominal ultrasound examination. (A,B) Hepatosplenomegaly with diffuse increased echogenicity of the hepatic parenchyma, consistent with hepatic steatosis. (C,D) Diffuse enlargement of the adrenal glands with lobulated echogenic foci producing posterior acoustic shadowing, compatible with parenchymal calcifications

**Figure 2:**
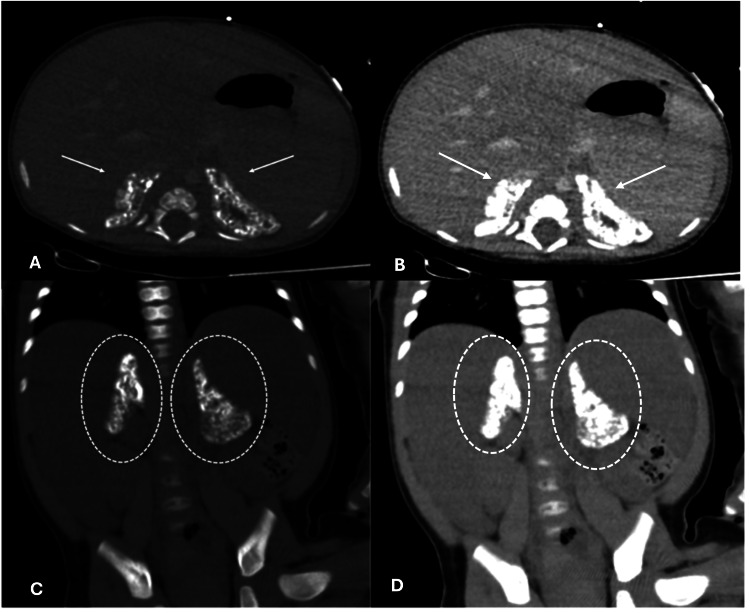
Selected contrast-enhanced abdominal computed tomography images displayed in soft-tissue and bone windows, in axial (A,B) and coronal (C,D) planes. Arrows and circles indicate the adrenal glands, which are enlarged and show punctate cortical calcifications while preserving their adreniform morphology

Given the suspicion of hepatomegaly, an abdominal ultrasound was performed, revealing posterior acoustic shadowing attributable to coarse calcifications of the adrenal glands (Figure 1). For further characterization, an abdominal computed tomography (CT) scan was obtained (Figure 2), demonstrating bilateral adrenal gland enlargement with coarse calcifications while preserving adrenal morphology.

In the context of suspected lysosomal storage disease (LAL deficiency: Wolman disease), genetic testing was performed, identifying a homozygous LIPA mutation c.652C>T p. (Arg218Ter), along with markedly reduced LAL activity (<0.03 mmol/PUNCH/HR; reference range: 0.37-2.30), thereby confirming the diagnosis. The patient is currently receiving enzyme replacement therapy, with a favorable clinical response, including weight gain and resolution of vomiting.

## Discussion

Wolman disease is a rare autosomal recessive inherited disorder caused by mutations in the LIPA gene, which encodes LAL, a key enzyme involved in the intracellular hydrolysis of triglycerides and cholesterol esters. The LIPA gene is located on the long arm of chromosome 10 (10q23.2-q23.3) and consists of 10 exons [1,2]. Multiple mutations associated with LAL deficiency have been described; those resulting in markedly reduced or absent enzymatic activity are specifically associated with Wolman disease [2].

A deficiency of LAL results in the progressive accumulation of triglycerides and cholesterol esters in multiple organs, particularly the liver, spleen, small intestine, and adrenal glands [3]. When LAL activity is reduced to less than 5% of normal levels, cholesterol release from lysosomes is significantly impaired, disrupting HDL synthesis and leading to a characteristic lipid profile marked by hypertriglyceridemia and elevated LDL levels, findings commonly observed in affected patients [4].

Bilateral adrenal gland calcification is a characteristic imaging finding in Wolman disease and is present in approximately 50% of cases [3]. This phenomenon is thought to result from overexpression of LDL receptors in adrenal cells secondary to defective lysosomal lipid degradation. Increased uptake of oxidized LDL leads to sustained intracellular calcium accumulation, causing progressive cellular injury and death, ultimately favoring glandular calcification [3,5].

Clinically, Wolman disease typically manifests in early infancy with hepatosplenomegaly, vomiting, and progression to liver failure. One of the most common initial manifestations is chronic diarrhea or steatorrhea due to gastrointestinal involvement, resulting in malabsorption and nutritional deterioration. The natural course of the disease includes accelerated atherosclerosis, progressive hepatic fibrosis evolving to cirrhosis, portal hypertension, and persistent gastrointestinal abnormalities [3,6].

Diagnosis is initially based on clinical suspicion; however, the presentation is nonspecific and may overlap with other metabolic or hepatic disorders. Laboratory studies typically reveal a characteristic lipid profile with elevated triglycerides and LDL levels accompanied by decreased HDL levels. Elevated liver transaminases, particularly alanine aminotransferase, are also frequently observed [2].

Diagnostic imaging plays a fundamental role in guiding the diagnosis. Plain abdominal radiography may demonstrate bilateral adrenal enlargement and calcification while preserving normal gland morphology. Abdominal ultrasound may reveal ascites, hepatomegaly with or without diffusely increased hepatic echogenicity, thickening of the intestinal walls, and bilateral adrenal calcifications. On CT, hepatic lipid infiltration manifests as an enlarged, hypodense liver, often associated with well-defined bilateral adrenal calcifications [7,8]. Definitive diagnosis is confirmed by identification of pathogenic mutations in the LIPA gene and measurement of LAL activity in lymphocytes or fibroblasts.

Differential diagnoses associated with adrenal calcifications include adrenal hemorrhage, infections such as tuberculosis, congenital adrenal hyperplasia, neuroblastoma, and other storage disorders such as Niemann-Pick disease. The latter is an inherited disorder of sphingomyelin metabolism characterized by accumulation in the liver, spleen, and central nervous system. Unlike Wolman disease, Niemann-Pick disease is not associated with malabsorption but presents with severe neurological involvement leading to early mortality [2].

Among these differential diagnoses, adrenal hemorrhage is the most common and is characterized by glandular size reduction with globular, irregular, and nonuniform calcifications. This morphology allows differentiation from the symmetric bilateral adrenal calcifications with preserved adreniform shape observed in Wolman disease [8].

This report presents the case of a female infant under two months of age with paraclinical and imaging findings consistent with Wolman disease. The lipid profile demonstrated hypertriglyceridemia and decreased HDL levels. Imaging studies revealed bilateral adrenal gland enlargement with preservation of adreniform morphology and calcifications, findings highly suggestive of this condition, along with hepatosplenomegaly demonstrated across multiple imaging modalities. The definitive diagnosis was confirmed by identification of a nonsense mutation in the LIPA gene.

Currently, there is no curative treatment for Wolman disease. Available therapeutic options aimed at reducing intracellular lipid accumulation include hematopoietic stem cell transplantation and enzyme replacement therapy, both of which have shown reductions in liver size and slowing of hepatic failure progression. Nevertheless, the disease remains associated with high mortality, underscoring the need for more effective therapeutic strategies [2,3].

## Conclusions

In infants with nonspecific gastrointestinal symptoms, failure to thrive, and hepatosplenomegaly, bilateral adrenal enlargement with coarse calcifications and preserved adreniform morphology on ultrasound or CT is highly suggestive of Wolman disease. Recognition of this characteristic finding enables the radiologist to raise early suspicion for a lysosomal storage disorder, recommend appropriate metabolic and genetic testing, and contribute to timely initiation of targeted therapy in a condition associated with high morbidity and mortality.
